# Social Determinants of Digital Health Adoption: Pilot Cross-sectional Survey

**DOI:** 10.2196/39647

**Published:** 2022-12-06

**Authors:** Sharvil Piyush Patel, Elizabeth Sun, Alec Reinhardt, Sanjaly Geevarghese, Simon He, Julie A Gazmararian

**Affiliations:** 1 Omnimed Inc Columbus, GA United States; 2 Emory University Atlanta, GA United States; 3 Rollins School of Public Health Emory University Atlanta, GA United States

**Keywords:** digital health, health accessibility, utilization, mobile health, mHealth, telemedicine

## Abstract

**Background:**

Interest in and funding for digital health interventions have rapidly grown in recent years. Despite the increasing familiarity with mobile health from regulatory bodies, providers, and patients, overarching research on digital health adoption has been primarily limited to morbidity-specific and non-US samples. Consequently, there is a limited understanding of what personal factors hold statistically significant relationships with digital health uptake. Moreover, this limits digital health communities’ knowledge of equity along digital health use patterns.

**Objective:**

This study aims to identify the social determinants of digital health tool adoption in Georgia.

**Methods:**

Web-based survey respondents in Georgia 18 years or older were recruited from mTurk to answer primarily closed-ended questions within the following domains: participant demographics and health consumption background, telehealth, digital health education, prescription management tools, digital mental health services, and doctor finder tools. Participants spent around 15 to 20 minutes on a survey to provide demographic and personal health care consumption data. This data was analyzed with multivariate linear and logistic regressions to identify which of these determinants, if any, held statistically significant relationships with the total number of digital health tool categories adopted and which of these determinants had absolute relationships with specific categories.

**Results:**

A total of 362 respondents completed the survey. Private insurance, residence in an urban area, having a primary care provider, fewer urgent emergency room (ER) visits, more ER visits leading to inpatient stays, and chronic condition presence were significantly associated with the number of digital health tool categories adopted. The separate logistic regressions exhibited substantial variability, with 3.5 statistically significant predictors per model, on average. Age, federal poverty level, number of primary care provider visits in the past 12 months, number of nonurgent ER visits in the past 12 months, number of urgent ER visits in the past 12 months, number of ER visits leading to inpatient stays in the past 12 months, race, gender, ethnicity, insurance, education, residential area, access to the internet, difficulty accessing health care, usual source of care, status of primary care provider, and status of chronic condition all had at least one statistically significant relationship with the use of a specific digital health category.

**Conclusions:**

The results demonstrate that persons who are socioeconomically disadvantaged may not adopt digital health tools at disproportionately higher rates. Instead, digital health tools may be adopted along social determinants of health, providing strong evidence for the digital health divide. The variability of digital health adoption necessitates investing in and building a common framework to increase mobile health access. With a common framework and a paradigm shift in the design, evaluation, and implementation strategies around digital health, disparities can be further mitigated and addressed. This likely will begin with a coordinated effort to determine barriers to adopting digital health solutions.

## Introduction

In the midst of the Digital Revolution and growing focus on health care access, the US health care system has begun to openly welcome digital health with an increased expansion of mobile apps, wearable electronic devices (eg, smartwatches and fitness trackers), artificial intelligence, and telemedicine [1]. Digital health and mobile health (mHealth) encompass the clinical application of information and communications technologies to improve health and wellness management [2]. In the context of the COVID-19 pandemic, interest and funding for digital health ventures reached US $24 billion in 2020, an increase from over US $5 billion in 2015 [3]. Not only is digital health increasingly used in clinical settings, but it is also prevalent in consumer settings in which individuals can access digital health products on everyday gadgets such as smartphones. A 2015 cross-sectional survey found that over half of mobile phone users had downloaded a health-related app [4]. Allowing individuals to manage their health on everyday devices has transformed the way that consumers interact with their health. Simultaneously, it has created a “divided digital revolution” due to the fact that many mHealth interventions may not be socioculturally developed and consequently exclude diverse groups [5].

With increasing interest from the Food and Drug Administration, Centers for Medicare and Medicaid Services, and other regulatory agencies and payers to bring mHealth into their care strategies and oversight, the potential for digital health solutions has evolved alongside consumer wellness offerings. Digital health is now inclusive of diagnostic, disease management, and clinical decision support tools [6]. New technologies aid health care providers by reducing the repetitiveness of their work and supporting their clinical decisions, workflow, and productivity [7]. In addition to digital health technology’s ability to standardize and improve the clinical experience, digital health’s value may be even greater as a mechanism to improve accessibility in a cost-effective manner [8].

Despite the potential benefits, digital health continues to lack a proper validation system for digital health interventions. This is a particularly pressing matter given the current landscape in which many technologies have been developed and as the implementation of new technology is shifting into a growing focus [9]. Many venture-funded digital health start-ups lack clinical robustness, once again indicating the need for increased emphasis on evidence-based approaches to product development [10]. Some researchers have called for validation domains spanning technical, clinical, and system validation [1]. While some literature exists on possible solutions that would increase the validation and utility of digital health tools, there exists a gap in digital health literature regarding uptake and use by the general population [1,11]. Moreover, much of the published literature supporting the increased implementation of digital health technology in improving patient outcomes is specific to certain patient populations and morbidities or was conducted outside the United States [1,12-16]. Such literature has provided valuable insight into the uptake of digital health tools. For example, one review focusing on older adults described 14 themes that affect the uptake of digital health, with the most common being technical literacy, lack of desire, and cost [17]. Another review focused on digital health interventions for adults with overweight and obesity highlighted attrition as a barrier to digital solutions and emphasized the need for digital health tools to be engaging for users [12]. The current specificity of research around the mHealth landscape not only provides useful information regarding digital health adoption but also indicates a need for a broader view of how individuals perceive and adopt digital health in the US health care system.

Fundamentally, empirical research in digital health and mHealth has a large gap in individual experience and adoption of broad digital health categories outside of condition-specific subgroups in the United States. Understanding the larger demographic trends and socioeconomic patterns in digital health uptake presents an important opportunity to numerically characterize the effect of social determinants within the burgeoning field of digital health. Ideally, the digital health innovation community can use original research about social determinants of digital health to think about and respond to the structural pillars of the digital health divide. In doing so, innovators can design mHealth interventions with an eye for accessibility. To achieve this goal, this study represents an initial, pilot investigation of the “social determinants of digital health” throughout the state of Georgia. The analysis statistically describes the use patterns of common digital health tools among web-based survey respondents in Georgia. By doing so, this investigation aims to understand how socioeconomic factors and care-seeking behaviors affect digital health uptake, and compares the adoption distribution across 8 common categories of digital health tools.

## Methods

### Study Design

This was a cross-sectional study in the area of digital health technology using survey data collected from June to November 2021. Participants responded to a web-based survey administered through REDCap that consisted of 172 possible questions (available upon request) to explore the use of and attitudes toward 8 common digital health tools. Common demographic questions and health care consumption questions were used as predictors of the social determinants of digital health use and were based on questions used in the National Health Interview Survey project [18]. Conditional and branching logic based on prior responses were used to pare down the questionnaire to deliver the right questions for the right participant.

### Study Population

Individuals were recruited from Amazon’s mTurk platform [19]. While patients were initially going to be recruited in a clinic setting, this became a safety hazard because of the COVID-19 pandemic and required the study team to use digital recruitment methods. In the context of this issue, mTurk provided access to a large heterogeneous population of willing research participants. The inclusion criteria required participants with existing mTurk worker accounts to be aged ≥18 years and be residents of the state of Georgia in the United States. Participants were compensated US $3 through mTurk’s internal platform. The data set began with 1022 records from which 630 duplicates, 29 incomplete responses, and 1 invalid survey were removed. Thus, the final analytic sample included 362 participants’ responses.

### Data Source

Survey questions were designed by the study team to investigate the use rates of different digital health tools and what factors may shape an individual’s use of digital health tools. The survey instrument contained primarily closed-ended question types with some opportunities to provide open-ended responses if the participant was willing. The questionnaire encompassed 172 possible questions in the English language that entailed the following domains: participant demographics and health consumption background, telehealth, digital health education, prescription management tools, digital mental health services, and doctor finder tools (available on request). The survey took approximately 15 to 20 minutes for participants to complete. Questions were presented to each participant in the same order. Demographic data and questions about health care use were also implemented to understand how social factors may impact digital health tool use.

Study data were collected and managed using REDCap electronic data capture tools hosted at Emory University [20,21]. REDCap is a secure web-based software platform designed to support data capture for research studies, providing an intuitive interface for validated data capture, audit trails for tracking data manipulation and export procedures, automated export procedures for seamless data downloads to common statistical packages, and procedures for data integration and interoperability with external sources.

### Data Measures

The analysis included 17 independent variables. There were 2 continuous independent variables: age (as reported by the respondent) and federal poverty level (calculated using household income and household size). There were 15 self-reported categorical variables: race, gender, ethnicity, insurance status, educational attainment, living area, access to the internet, access to health care, primary care–seeking behavior in non–life-threatening events (as defined by the type of clinic visited during said events), having a routine primary care provider (PCP), amount of PCP visits in the last 12 months, amount of emergency room (ER) visits for nonurgent events in the last 12 months, amount of ER visits for urgent events in the last 12 months, amount of ER visits leading to inpatient stays in the last 12 months, and presence of chronic conditions (actual survey items available on request).

There was 1 continuous dependent variable (the number of digital health tool categories used by a patient) and 8 yes or no questions for self-reported digital health tool use in the following categories: telehealth, digital health education, prescription management tools, doctor finders, social services referral tools, digital mental health tools, digital insurance navigators, and patient portals.

### Statistical Analysis

First, descriptive statistics were calculated to determine the frequency of using the 8 digital health categories. Second, an adjusted linear regression was completed between the independent variables and the number of digital health tool categories adopted by a respondent. Third, separate logistic regressions were used to identify predictors of use for each category of tools. Statistical significance was determined by *P* values <.05. All statistical analyses were completed using R version 4.0.5 (R Foundation for Statistical Computing) with the packages tidyverse, MASS, and mice [22-25]. Multiple imputation based on mean-matching was used to impute missing data within the sample. Imputation required at least 50% or more of the existing data, and all of the included independent and dependent variables required some imputation.

### Ethical Considerations

This study was approved by Emory University’s institutional review board (reference STUDY00001999). Participants were recruited from Amazon’s mTurk platform and were compensated US $3 for their participation through mTurk’s internal system. All participation was voluntary, and no participant was subject to any harm. Informed consent was obtained from each participant regarding what their involvement in the study entailed and how their responses would be handled. The privacy of research participants was maintained throughout the study, and all responses were deidentified.

## Results

Over half (n=189, 52.2%) of the 362 respondents reported having private insurance, and more than half (n=160, 60.2%) of them had earned at least a bachelor’s degree (Table 1). Almost all (n=302, 98.6%) respondents reported always or almost always having reliable access to the internet. About one-third (n=118, 34.7%) of the respondents reported having at least one chronic condition. On average, respondents used more than 3 digital health tools.

**Table 1:** Descriptive statistics of the respondents presented as percentages, means, or medians (n=362).

Variables		Value
**Age (years)**		
	Mean (SD)	37 (0.6)
	Median (range)	34 (18-73)
Federal poverty line ratio, mean (SD)		3 (0.28)
**Number of PCP^a^ visits in the past 12 months**		
	Mean (SD)	4 (0.14)
	Median (range)	3 (1-11)
**Number of ER^b^ visits for nonurgent issues in the past 12 months**		
	Mean (SD)	2 (0.1)
	Median (range)	1 (1-10)
**Number of ER visits for urgent issues in the past 12 months**		
	Mean (SD)	2 (0.11)
	Median (range)	1 (1-11)
**Number of ER visits leading to inpatient stay**		
	Mean (SD)	4 (0.05)
	Median (range)	1 (1-10)
**Difficulty accessing health care (Likert scale: 1 is very hard, 5 is very easy)**		
	Mean (SD)	4 (0.05)
	Median (range)	4 (1-5)
**Seek care in non–life-threatening situations, n (%)**		
	Primary care physician	191 (52.8)
	Urgent care	111 (30.7)
	Emergency room	30 (8.3)
	Other	30 (8.3)
**Has PCP, n (%)**		
	Yes	275 (76.0)
	No	87 (24.0)
**Presence of chronic condition, n (%)**		
	Yes	118 (34.7)
	No	222 (61.3)
	No response	22 (6.0)
**Race, n (%)**		
	Non-White	104 (28.7)
	White	253 (65.3)
	No response	5 (1.4)
**Gender, n (%)**		
	Male	218 (60.9)
	Female	140 (39.1)
	No response	4 (1.1)
**Ethnicity, n (%)**		
	Hispanic	32 (8.8)
	Not Hispanic	322 (89.0)
	No response	8 (2.2)
**Insurance, n (%)**		
	Public insurance	95 (26.2)
	Private insurance	189 (52.2)
	A mix of both public and private insurance	23 (6.4)
	I have insurance, but I’m not sure what type	8 (2.2)
	Uninsured	47 (13.0)
**Education, n (%)**		
	High school or less	27 (7.5)
	Some college, no degree	85 (23.5)
	Associate’s degree	32 (8.8)
	Bachelor’s degree	160 (44.2)
	Master’s degree	49 (13.5)
	Professional/doctoral degree	9 (2.5)
**Living area, n (%)**		
	Urban	104 (28.7)
	Suburban	187 (51.7)
	Rural	71 (19.6)
**Internet access, n (%)**		
	Always have access	302 (83.4)
	Does not always have access	55 (15.2)
	No response	5 (1.4)
**Number of digital health tool categories adopted**		
	Mean (SD)	4 (0.11)
	Median (range)	4 (0-8)
**Telehealth use, n (%)**		
	No	140 (38.7)
	Yes	222 (61.3)
**Health education tool use, n (%)**		
	No	124 (34.3)
	Yes	238 (65.7)
**Prescription management tool use, n (%)**		
	No	232 (64.1)
	Yes	130 (35.9)
**Doctor finder tool use, n (%)**		
	No	185 (51.1)
	Yes	177 (48.9)
**Social service referral tool use, n (%)**		
	No	289 (79.8)
	Yes	73 (20.2)
**Mental health tool use, n (%)**		
	No	239 (66.0)
	Yes	123 (34.0)
**Insurance management tool use, n (%)**		
	No	239 (66.0)
	Yes	123 (34.0)
**Patient portal use, n (%)**		
	No	123 (34.0)
	Yes	239 (66.0)

^a^PCP: primary care provider.

^b^ER: emergency room.

Results from the linear regression analysis indicate that for total digital tool adoption count, the model was statistically significant (*P*<.001; adjusted *R*^2^=0.28; Table 2). Six variables were statistically significant at the α<.05 level. On average, respondents with private insurance used 0.55 more digital health tools than respondents with public insurance (*P*=.03). Respondents living in rural or suburban areas used 0.75 fewer digital health tools than respondents in urban areas (*P*=.003 and .02, respectively). Patients without a PCP used 1.25 fewer digital health tools than respondents with a PCP (*P*=.005), while respondents with no chronic condition used 0.73 fewer tools than respondents with chronic conditions (*P*=.001). A 1-unit increase in the number of urgent ER visits was associated with a 0.4 decrease in digital health tools used (*P*=.04), but a 1-unit increase in the number of ER visits leading to inpatient stays resulted in the use of 0.64 more digital health tools (*P*=.002), on average. No multicollinearity was observed.

None of the predictors held a statistically significant relationship with the outcomes across all 8 logistic regression models (Multimedia Appendix 1). In fact, the total number of significant variables across each logistic regression model varied greatly, with an average of 3.5 statistically significant predictors in a model. For example, the logistic regression model for the doctor finder only had a significant relationship with 1 variable (residential area), whereas telehealth use had 6 significant predictors (number of PCP visits in the past 12 months, self-reported race, insurance status, residential area, PCP status, and chronic condition status).

**Table 2:** Estimates for the linear regression model on the total number of digital health tool categories adopted (n=362).

Variables			β (95% CI)		*P* value
Intercept			4.08 (3.06 to 5.1)		<.001
Age			–0.06 (–0.24 to 0.12)		.49
Federal poverty level			–0.01 (–0.18 to 0.17)		.96
Number of PCP^a^ visits in past 12 months			0.05 (–0.21 to 0.31)		.71
Number of nonurgent ER^b^ visits in past 12 months			0.31 (0 to 0.63)		.05
Number of urgent ER visits in past 12 months			–0.31 (–0.67 to 0.04)		.08
Number of ER visits leading to inpatient stays in past 12 months			0.58 (0.2 to 0.95)		<.001
Difficulty			–0.02 (–0.21 to 0.18)		.87
**Seek care in non–life-threatening situations**					
	Primary care physician (reference)	N/A^c^		N/A	
	Urgent care	0.42 (–0.01 to 0.85)		.06	
	Emergency room	0.28 (–0.43 to 0.98)		.44	
	Other	0.51 (–0.19 to 1.21)		.15	
**Has PCP**					
	Has PCP (reference)	N/A		N/A	
	No PCP	–0.61 (–1.73 to 0.05)		.07	
**Presence of chronic condition**					
	Has chronic condition (reference)	N/A		N/A	
	No chronic condition	–0.72 (–1.1 to –0.33)		<.001	
**Race**					
	Non-White (reference)	N/A		N/A	
	White	0.20 (–0.2 to 0.59)		.34	
**Gender**					
	Female (reference)	N/A		N/A	
	Male	–0.38 (–0.76 to 0)		.05	
**Ethnicity**					
	Hispanic (reference)	N/A		N/A	
	Non-Hispanic	0.00 (–0.59 to 0.58)		.99	
**Insurance**					
	Public (reference)	N/A		N/A	
	Private	0.37 (–0.09 to 0.83)		.11	
	Mix	0.24 (–0.53 to 1.01)		.54	
	Unsure	–0.18 (–1.41 to 1.06)		.78	
	Uninsured	–0.52 (–1.21 to 0.18)		.14	
**Education**					
	Professional degree (reference)	N/A		N/A	
	Master’s	–0.29 (–1.57 to 0.98)		.65	
	Bachelor’s	–0.20 (–1.01 to 0.61)		.63	
	Associate’s	–0.18 (–0.88 to 0.51)		.60	
	Some college	–0.23 (–1.09 to 0.63)		.60	
	Less than high school or high school graduate/GED^d^	–0.65 (–1.37 to 0.08)		.08	
**Living area**					
	Urban (reference)	N/A		N/A	
	Suburban	–0.61 (–1.06 to –0.16)		.01	
	Rural	–0.62 (–1.17 to –0.06)		.03	
**Internet**					
	Always has access (reference)	N/A		N/A	
	Does not always have access	–0.25 (–0.76 to 0.25)		.32	

^a^PCP: primary care provider.

^b^ER: emergency room.

^c^N/A: not applicable.

^d^GED: General Educational Development.

## Discussion

### Principal Findings

Given the paucity of literature surrounding the use of common digital health tools by the general population in the United States, this paper is a novel investigation on how 8 common forms of digital health management tools are adopted and used in a diverse population in Georgia. Surprisingly, there are large variations in how many and which factors predict digital health use uptake. The large variability indicates that there is likely diversity in the populations using various types of digital health tools. Moreover, this analysis’ emphasis on the variability in the adoption of digital health potentially highlights a gap between mHealth’s ideal and actual implementations; this is especially true when relationships that would logically be meaningful did not appear in the analysis. Ideally, mHealth would be a tool that allows medically indigent populations to improve their access to health care and to community-based resources that empower their care navigation efforts. Specifically, these findings highlight that digital health innovations are not always distributed equitably or to the people who would ostensibly need it the most. For example, the use of social services referral tools did not vary with education level, federal poverty level ratio, or lack of insurance. Ideally, socioeconomically disadvantaged populations—proxied for by education level, federal poverty level, and insurance status—would have significantly higher rates of adoption for social services referral tools since these tools are meant to expand this population’s access to social services. Similarly, digital prescription management tools had no statistically significant relationship with insurance status, which levies a similar concern since patients who are uninsured should be more likely to use digital tools to lower out-of-pocket prescription costs when holding the chronic condition status constant. Fundamentally, this initial investigation spotlights the need for incorporating more real-world evidence into digital health distribution, use, and outcomes beyond the controlled trial environment.

Interventions can and should be targeted to reach patients that traditionally cannot access health care options; however, these results indicate that, in practice, the patients with the greatest need may not find these technologies accessible. If this is evidence that the most socially and medically indigent patients cannot access new innovations, inequality can either become a substantial barrier to improving health outcomes at scale or even exacerbate health disparities across a digital divide. With the incorporation of on-the-ground use data, innovators and researchers can assess whether inequalities in uptake are a result of the inequalities in patient access to prerequisite infrastructure (eg, smartphones, Wi-Fi, or computers) or if some current investigations of digital health interventions may simply lack external validity, thus requiring a paradigm shift in the design, evaluation, and implementation strategies around digital health. These observations align with the fundamental shift in health intervention evaluation methodologies. More specifically, there is an increasing emphasis on generating and using real-world evidence and data to measure digital health efficacy [26].

These results functionally support the extant literature’s depiction of digital health inequality across various digital health domains and diverse populations. For example, Tappen et al [27] described a “deep digital health divide” in older populations that is associated with age, education, income, and ethnicity. Likewise, Saeed and Masters [28] noted that in psychiatric conditions, telehealth use is negatively correlated with lower socioeconomic status. Moreover, Brown et al [16] found that significant disparities exist in access to telemedical care among cardiovascular patients that are low-income, older adults, or Black or Hispanic [16]. Perhaps unsurprisingly, historic drivers of inequities in health care continue to exist in a similar manner in the digital health space. The impact of social determinants of health goes beyond access to medical care and pure health outcomes. There is an apparent impact on how the social determinants can affect access to digital health technologies, which may be a mediating variable for health outcomes and access to care. Fortunately, there is a movement toward prioritizing contextually tailored mHealth interventions that can mitigate inequalities in access to digital health [5].

### Strengths and Limitations

The self-reported data collected from this investigation has at least three strengths. First, the large sample size provided minimizes concerns about response bias and improves statistical strength. Moreover, the use of mTurk to recruit respondents allows for a much larger pool than the original target population, focusing on patient-defined populations. As a result, this study included a more diverse panel of respondents. Second, the survey examined data across a broad array of digital health tools rather than focusing on one specific category of tools. As a result, this novel data set provides a broad view of digital health uptake across multiple subsets of patients. This also provides better comparisons across the various classes of commercialized technology. Third, the variety of independent predictors allows for better adjustment in the linear and logistic regression models to control for confounding variables’ bias.

Despite these strengths, there are at least three limitations. First, respondents were recruited and surveyed using a web-based platform (mTurk), which inherently introduces the effects of self-selection. For instance, this study analyzes how digital (ie, internet-connected and internet-enabled) health tools are used by asking questions to respondents who therefore are adept with technology and have substantial access to digital platforms before the survey. Second, the platform does not provide the opportunity to randomly sample respondents, limiting generalizability. Third, the cross-sectional nature of the data and analysis precludes causal induction. This analysis cannot link respondent behavior over time with the changes in their independent predictors’ statuses. In the future, semicausal and causal methods should be used to establish cause-and-effect relationships.

### Future Directions

Further work to identify and reduce barriers to entry for digital health tools is vital to expanding its impact and promoting equity with the advent of new technologies. Understanding how digital health disseminates will be crucial to intervention design, implementation, and evaluation. The existing variability in the adoption of mHealth underscores the lack of a common framework to increase mHealth use across diverse patient populations, which ultimately limits an intervention’s potential for success. Innovative strategies can help improve access moving forward; however, this will require digital health innovators and regulators to regularly collect and review data regarding mHealth adoption. This can help evaluate the social and clinical returns on funding for digital health interventions, especially those aimed at improving health care access.

The large variability in adoption across digital health tools indicates that a one-size-fits-all approach to deploying these tools will likely mitigate their potential impact. Consequently, additional research is needed to better understand these patterns at larger scales and across more diverse populations, and the full range of factors that contribute to intervention uptake. Similarly, the digital health innovation community should rebuild the evaluation framework for evaluating tool distribution and adoption throughout the intervention’s lifecycle to ensure that patients are able to equitably access these services. More specifically, special consideration should be given to how new tools can contribute to the digital health divide, so when new tools are deployed, innovators should look at the characteristics of users to determine if adoption is equitably distributed.

### Conclusions

This investigation establishes an initial portrait of how variable the use of digital health tools is across various patient demographics. Moreover, these results indicate that populations who could benefit the most from using certain tools (eg, patients who are socioeconomically disadvantaged and who would benefit from a social services referral tool) are not using these technologies. Although these tools are already publicly available, populations that could realize substantial benefits from this technology experience larger barriers to entry and sustained use (eg, information, internet, and cost obstacles). Ultimately, while these tools can be valuable, user uptake is the most important prerequisite to clinical and social utility.

## Appendix

Multimedia Appendix 1 Estimates for the logistic regression models of use of tools in digital health categories (n=362).

## Abbreviations

ER: emergency room
mHealth: mobile health
PCP: primary care provider
