# Supplementary material for: Social Determinants of Digital Health Adoption: Pilot Cross-sectional Survey
Source: JMIR Form Res. 2022 Dec 6;6(12):e39647. doi: 10.2196/39647 (PMC9768657; doi:10.2196/39647)
Supplement: Multimedia Appendix 1 [file formative_v6i12e39647_app1.pdf]

Multimedia Appendix 1. Estimates for the logistic regression models of use of tools in digital health categories (n=362).

Variates	Odds Ratio (95% CI)							
	**Digital Health Education**	**Prescription Management**	**Doctor Finder**	**Social Services Referral**	**Mental Health**	**Insurance Management**	**Patient Portal**	**Telehealth**
**Intercept**	5.72 (1.28, 25.56)	1.77 (0.39, 7.94)	1.52 (0.38, 6.06)	0.65 (0.11, 3.90)	0.95 (0.21, 4.36)	1.31 (0.31, 5.60)	2.56 (0.57, 11.42)	4.84 (1.02, 22.83)
**Age**	0.97 (0.76, 1.25)	0.98 (0.76, 1.26)	0.81 (0.64, 1.02)	1.21 (0.88, 1.66)	0.87 (0.67, 1.14)	1.01 (0.78, 1.31)	0.96 (0.75, 1.23)	0.83 (0.64, 1.07)
**Federal Poverty Level**	1.31 (1.00, 1.73)	1.01 (0.78, 1.29)	1.02 (0.81, 1.28)	0.85 (0.59, 1.23)	0.96 (0.74, 1.24)	0.80 (0.60, 1.07)	0.92 (0.72, 1.17)	0.88 (0.68, 1.13)
**Number of PCP Visits in Past 12 Months**	1.26 (0.80, 2.00)	1.43 (0.92, 2.22)	0.82 (0.54, 1.25)	0.91 (0.51, 1.62)	1.14 (0.73, 1.79)	1.04 (0.66, 1.65)	1.57 (0.97, 2.54)	2.82 (1.61, 4.93)
**Number of Non-Urgent ER Visits in Past 12 Months**	1.01 (0.64, 1.61)	0.88 (0.54, 1.44)	1.41 (0.90, 2.20)	1.41 (0.88, 2.26)	1.36 (0.87, 2.10)	1.42 (0.91, 2.24)	1.13 (0.73, 1.76)	1.28 (0.73, 2.26)
**Number of Urgent ER Visits in Past 12 Months**	0.50 (0.29, 0.86)	0.84 (0.51, 1.39)	0.65 (0.39, 1.06)	0.83 (0.49, 1.41)	0.72 (0.43, 1.20)	0.89 (0.54, 1.47)	1.21 (0.74, 1.99)	0.66 (0.36, 1.19)
**Number of ER Visits Leading to Inpatient Stays in Past 12 Months**	2.15 (1.18, 3.90)	1.91 (1.10, 3.31)	1.61 (0.93, 2.78)	1.77 (1.01, 3.09)	2.16 (1.22, 3.82)	1.29 (0.76, 2.20)	0.69 (0.42, 1.15)	1.33 (0.69, 2.55)
**Race**								
White	0.85 (0.49, 1.48)	1.65 (0.90, 3.01)	1.41 (0.83, 2.39)	1.08 (0.50, 2.33)	0.90 (0.50, 1.63)	0.85 (0.47, 1.54)	1.76 (1.02, 3.03)	2.17 (1.23, 3.83)
**Gender**								
Male	0.67 (0.39, 1.15)	0.53 (0.30, 0.95)	0.95 (0.58, 1.57)	0.90 (0.44, 1.83)	0.34 (0.19, 0.63)	1.59 (0.92, 2.75)	0.82 (0.48, 1.40)	0.93 (0.53, 1.63)
**Ethnicity**								
Non-Hispanic	0.79 (0.33, 1.86)	0.53 (0.23, 1.21)	0.61 (0.28, 1.33)	1.50 (0.55, 4.06)	1.82 (0.74, 4.46)	1.22 (0.53, 2.83)	1.14 (0.52, 2.51)	0.62 (0.25, 1.50)
**Insurance**								
Private insurance	1.40 (0.73, 2.69)	0.96 (0.49, 1.87)	1.83 (0.99, 3.39)	0.44 (0.20, 0.95)	1.72 (0.86, 3.44)	0.90 (0.47, 1.71)	3.30 (1.72, 6.33)	2.71 (1.33, 5.53)
Mix of public and private	1.01 (0.34, 2.97)	1.13 (0.38, 3.36)	0.86 (0.31, 2.35)	2.11 (0.68, 6.53)	2.13 (0.72, 6.33)	0.79 (0.28, 2.23)	0.98 (0.37, 2.65)	1.14 (0.34, 3.80)
Insurance but unsure	0.57 (0.23, 1.42)	1.83 (0.71, 4.72)	0.57 (0.23, 1.43)	0.45 (0.13, 1.53)	0.97 (0.35, 2.70)	0.18 (0.05, 0.64)	1.13 (0.47, 2.68)	1.09 (0.42, 2.80)
**Education**								
High school graduate/GED	0.88 (0.33, 2.33)	0.78 (0.27, 2.22)	0.54 (0.21, 1.40)	0.41 (0.10, 1.65)	0.76 (0.26, 2.22)	0.35 (0.13, 0.99)	0.60 (0.20, 1.81)	1.05 (0.38, 2.92)
Some college, no degree	2.23 (0.66, 7.60)	0.55 (0.15, 1.97)	0.93 (0.30, 2.85)	1.16 (0.25, 5.31)	0.54 (0.14, 2.03)	0.58 (0.17, 1.93)	0.76 (0.20, 2.82)	1.34 (0.39, 4.66)
Associate's degree	1.73 (0.67, 4.45)	1.03 (0.38, 2.83)	0.75 (0.30, 1.84)	0.67 (0.18, 2.41)	1.18 (0.42, 3.28)	0.62 (0.24, 1.58)	0.43 (0.15, 1.26)	0.93 (0.35, 2.50)
Bachelor's degree	2.47 (0.78, 7.84)	0.96 (0.30, 3.04)	0.75 (0.26, 2.15)	0.55 (0.12, 2.41)	1.09 (0.34, 3.54)	0.67 (0.22, 2.03)	0.46 (0.13, 1.57)	2.83 (0.81, 9.93)
Master's degree	0.95 (0.17, 5.15)	0.22 (0.02, 2.69)	0.69 (0.13, 3.61)	0.00 (0.00, 0.00)	2.70 (0.47, 15.70)	1.17 (0.20, 6.79)	0.43 (0.07, 2.54)	0.92 (0.16, 5.26)

**Living Area**								
Suburban	0.77 (0.40, 1.48)	0.53 (0.28, 1.02)	0.93 (0.51, 1.69)	0.44 (0.20, 0.94)	0.37 (0.19, 0.70)	0.54 (0.28, 1.03)	1.14 (0.61, 2.14)	0.39 (0.19, 0.80)
Rural	1.09 (0.49, 2.45)	0.79 (0.36, 1.73)	0.46 (0.22, 0.98)	0.38 (0.14, 1.05)	0.46 (0.21, 1.02)	0.55 (0.24, 1.23)	0.89 (0.40, 1.94)	0.44 (0.19, 1.04)
**Internet**								
Access most of the time	0.55 (0.28, 1.09)	1.57 (0.79, 3.14)	1.20 (0.62, 2.32)	1.32 (0.59, 2.96)	0.61 (0.29, 1.28)	1.06 (0.52, 2.18)	0.51 (0.26, 0.99)	1.76 (0.83, 3.76)
**Difficulty Accessing Healthcare**	0.93 (0.71, 1.23)	0.96 (0.73, 1.26)	1.04 (0.80, 1.35)	0.86 (0.61, 1.21)	0.78 (0.59, 1.04)	1.14 (0.84, 1.54)	1.08 (0.83, 1.41)	1.06 (0.79, 1.41)
**Usual Place to Seek Care**								
Urgent care	1.36 (0.73, 2.52)	2.64 (1.41, 4.94)	1.23 (0.70, 2.17)	1.21 (0.55, 2.63)	0.83 (0.44, 1.55)	1.22 (0.66, 2.26)	1.57 (0.85, 2.90)	1.09 (0.58, 2.04)
Emergency room	0.93 (0.34, 2.52)	1.11 (0.38, 3.27)	1.33 (0.50, 3.57)	1.99 (0.62, 6.32)	0.93 (0.32, 2.68)	1.48 (0.51, 4.36)	1.42 (0.54, 3.70)	0.87 (0.29, 2.57)
Other	1.83 (0.67, 4.95)	2.34 (0.88, 6.21)	1.44 (0.57, 3.61)	2.18 (0.60, 7.94)	0.50 (0.16, 1.55)	1.09 (0.35, 3.42)	2.46 (0.89, 6.80)	0.90 (0.34, 2.40)
**PCP Status**								
No PCP	0.59 (0.18, 1.89)	0.17 (0.05, 0.55)	1.28 (0.44, 3.69)	1.00 (0.23, 4.28)	0.72 (0.23, 2.29)	0.90 (0.28, 2.90)	0.17 (0.05, 0.56)	0.05 (0.01, 0.21)
**Chronic Condition Status**								
No Chronic Condition	0.32 (0.17, 0.59)	0.54 (0.31, 0.93)	0.79 (0.46, 1.34)	0.58 (0.28, 1.20)	0.80 (0.45, 1.44)	0.82 (0.45, 1.50)	0.61 (0.34, 1.09)	0.54 (0.30, 0.998)
